# The Potential Application of Magnetic Nanoparticles for Liver Fibrosis Theranostics

**DOI:** 10.3389/fchem.2021.674786

**Published:** 2021-05-14

**Authors:** Aziz Eftekhari, Allahveirdy Arjmand, Ayyub Asheghvatan, Helena Švajdlenková, Ondrej Šauša, Huseyn Abiyev, Elham Ahmadian, Oleh Smutok, Rovshan Khalilov, Taras Kavetskyy, Magali Cucchiarini

**Affiliations:** ^1^ Maragheh University of Medical Sciences, Maragheh, Iran; ^2^ Polymer Institute, Slovak Academy of Sciences, Bratislava, Slovakia; ^3^ Russian Institute for Advanced Study, Moscow State Pedagogical University, Moscow, Russian Federation; ^4^ Department of Surface Engineering, The John Paul II Catholic University of Lublin, Lublin, Poland; ^5^ Institute of Physics, Slovak Academy of Sciences, Bratislava, Slovakia; ^6^ Department of Nuclear Chemistry, Faculty of Natural Sciences, Comenius University in Bratislava, Bratislava, Slovakia; ^7^ Department of Biochemistry, Azerbaijan Medical University, Baku, Azerbaijan; ^8^ Kidney Research Center, Tabriz University of Medical Sciences, Tabriz, Iran; ^9^ Department of Chemistry and Biomolecular Science, Clarkson University, Potsdam, NY, United States; ^10^ Institute of Cell Biology, National Academy of Sciences of Ukraine, Lviv, Ukraine; ^11^ Department of Biophysics and Biochemistry, Baku State University, Baku, Azerbaijan; ^12^ Institute of Radiation Problems, National Academy of Sciences of Azerbaijan, Baku, Azerbaijan; ^13^ Department of Biology and Chemistry, Drohobych Ivan Franko State Pedagogical University, Drohobych, Ukraine; ^14^ Center of Experimental Orthopaedics, Saarland University Medical Center, Homburg, Germany

**Keywords:** liver fibrosis, magnetic nanoparticles, nanomedicine, theranostics, drug delivery

## Abstract

Liver fibrosis is a major cause of morbidity and mortality worldwide due to chronic liver damage and leading to cirrhosis, liver cancer, and liver failure. To date, there is no effective and specific therapy for patients with hepatic fibrosis. As a result of their various advantages such as biocompatibility, imaging contrast ability, improved tissue penetration, and superparamagnetic properties, magnetic nanoparticles have a great potential for diagnosis and therapy in various liver diseases including fibrosis. In this review, we focus on the molecular mechanisms and important factors for hepatic fibrosis and on potential magnetic nanoparticles-based therapeutics. New strategies for the diagnosis of liver fibrosis are also discussed, with a summary of the challenges and perspectives in the translational application of magnetic nanoparticles from bench to bedside.

## Introduction

The unique intrinsic regenerative properties of the liver have long been known, reaching up to 70% in healthy liver tissue (Michalopoulos and Bhushan, 2021). In fact, it is the only organ with the capability of replacing potentially injured cells via regeneration processes in humans (Michalopoulos and Bhushan, 2021). However, in spite of such a feature, the prevalence of hepatic diseases exhibits an upward trend with a high rate of mortality.

Recurrent and chronic injuries may lead to liver fibrosis which frequently leads to cancer, with an enhanced accumulation of extracellular matrix (ECM) components like type-I and -III collagen, resulting in scar deposition and triggering the disease severity (Fan et al., 2020). The continuous accumulation of ECM disturbs several key functions mediated by the liver like detoxification and results in disturbances in the hepatic blood flow. Moreover, fibrosis is associated with the recruitment of inflammatory cytokines that can augment tumor development (Stolfi et al., 2021). If left untreated, cirrhosis may occur subsequently to fibrosis which is connected with portal hypertension, liver failure, hepatic encephalopathy, as well as the elevated risk of hepatocellular carcinoma (HCC) (Stolfi et al., 2021). Any stimulus that can disturb hepatic-sensitive cells can result in liver injury. Genetic factors and secondary liver damage due to the presence of other diseases such as cholestatic disorders, viral hepatitis, autoimmune diseases, and chronic alcohol abuse can also induce liver fibrosis. Nonalcoholic steatohepatitis (NASH) and nonalcoholic fatty liver disease (NALFD) stand for the main etiologies of the disease. Obesity and demographic alterations resulting from aging also increased the prevalence of hepatic fibrosis (Nascimbeni et al., 2020). NAFLD is considered the major cause of chronic liver diseases and the top indication of liver transplantation (Mikulic and Mrzljak, 2020). Other factors such as the infiltration of inflammatory cells can promote the progression of NASH, NAFLD, and fibrosis. The severity of fibrosis has been connected with the mortality associated with liver diseases as demonstrated in numerous longitudinal clinical investigations. Consistently, the efficiency for the assessment of pharmacological options against NAFLD is their effect on liver fibrosis that might also exert a positive outcome on non-hepatic disorders (Polyzos et al., 2020). Experts estimate that the mortality rate of liver-related diseases will significantly rise in the upcoming years (Mikulic and Mrzljak, 2020). Fibrosis may be characterized a dysregulated wound healing process, resulting in the generation of tissue scars (Simkin et al., 2020). The presence of bridging fibers between portal regions is the signs of advanced stages of liver fibrosis (Chang and Li, 2020).

The inability of conventional treatments to deliver adequate concentrations of drugs into the liver has restricted their application. Targeted delivery of pharmaceutics to the liver tissue via the application of nanotechnology has grabbed great attention in the recent years (Mikulic and Mrzljak, 2020; Nascimbeni et al., 2020; Polyzos et al., 2020). Different types nanoparticle systems have been utilized in the treatment of liver fibrosis. The unique specifications of these systems are due to their size, shape, structure and surface features. Polymers, liposomes, and distinct moieties have been used as nanomedicine formulations in the treatment of hepatic diseases. These systems have efficiently delivered different therapeuticall agents to the liver cells.

Magnetic nanoparticles (MNPs) are promising nanomaterials with a wide range of applications in drug delivery, tissue engineering, and magnetic resonance imaging (MRI) due to their superparamagnetic effects. The use of inorganic NPs has been an appealing strategy to carry and deliver drugs to the special tissues. A metal and/or a metal oxide core covered with an organic layer is the characterization of these particles which provides unique electrical, optical and magnetic properties. The current review summarizes potential targets and the application of emerging MNPs for theranostic purposes against hepatic fibrosis. Approaches in the implementation of MNPs also discussed.

## Molecular Mechanism of Liver Fibrosis and Recent Clinical Trials

**FIGURE 1:**
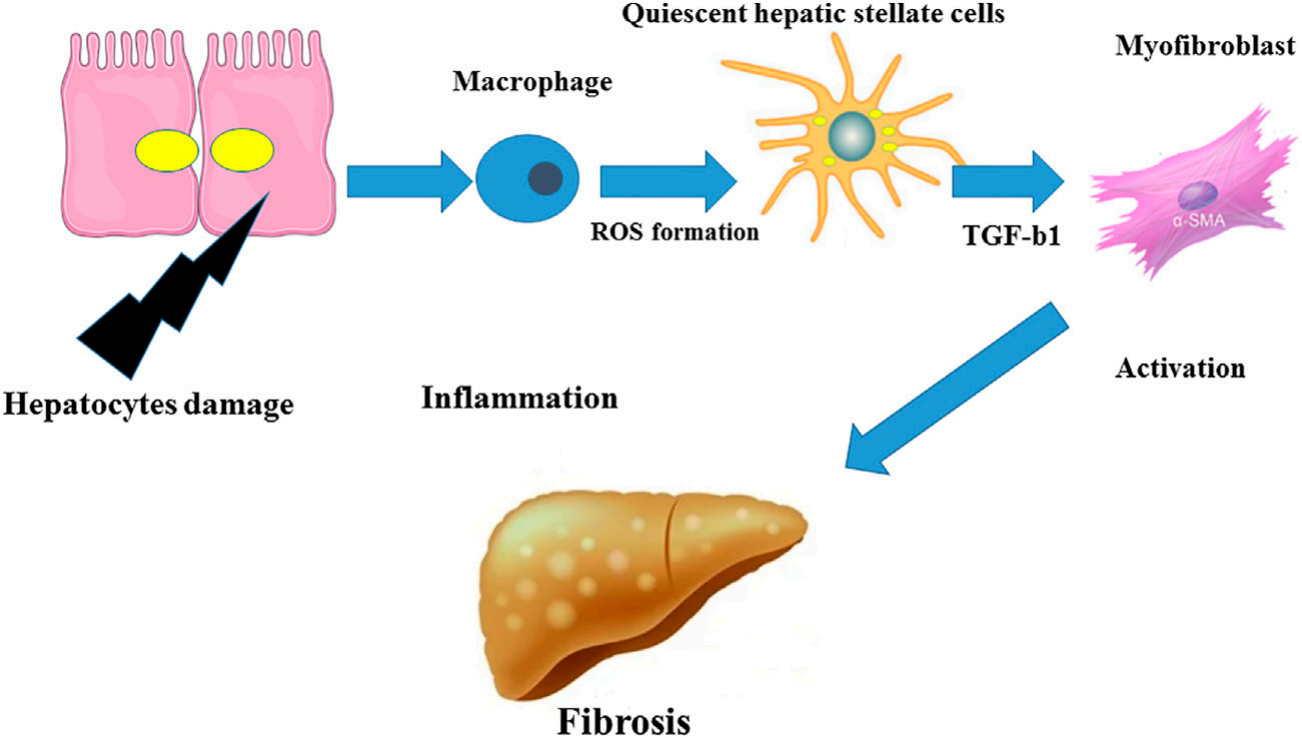
Inflammation in fibrosis. The inflammatory response could be induced by hepatocyte damage leading to the activation of macrophages and to a production of ROS and TGF-β1 and to the differentiation of quiescent HSCs in MFBs which in turn may trigger liver fibrosis.

Fibrogenesis, the process of fibrosis development, can be assessed from the cellular point of view. Zajicek and coworkers (Zajicek et al., 1985) first reported that hepatocyte regeneration occurs in a streaming fashion. The gradual streaming of hepatocytes located at the portal space towards the hepatic vein occurs and then the apoptotic machinery removes these cells, a process lasting about 200 days in rats (Zajicek et al., 1985). During the progression of liver fibrosis, hepatic stellate cell (HSC)-derived ECM fills the empty spaces resulting from the traveling of hepatocytes. In healthy subjects, fresh hepatocytes replenish these spaces. Hepatocytes secrete molecules which act as danger signals for other cell types during the apoptosis process (Jiang et al., 2021). These signals activate immune cells which further release other factors that trigger the apoptosis of liver cells (Figure 1). All these processes surge the fibrogenic response. The initiation of the inflammatory response in NAFLD is evoked by innate immune cells which express pattern recognition receptors (PRR) to sense inflammatory mediators and danger-/pathogen-associated molecular pattern molecules (DAMP, PAMP, respectively) (Sosa et al., 2020). Recent investigations clarified the role of cholangiocytes in liver diseases especially in cholestatic liver injury (Zhou, 2020). The proliferation of cholangiocytes has been considered as an important inducer of liver fibrosis in biliary atresia (Chusilp et al., 2020). A long non-coding RNA has been identified to provoke this proliferation and thus may represent a potential target to treat fibrosis (Xiao et al., 2019). The production of bile acids decreases as a result of cholangiocyte injuries through toxic liver damages. Extracellular vesicles (EVs) and microRNAs are pivotal factors in regulating the metabolism of cyclic adenosine monophosphate (cAMP) in cholangiocytes (Sato et al., 2018). In addition to cholangiocytes, mast cells were implicated in the development of liver fibrosis. Mast cell-deficient animals have lower degrees of liver fibrosis. The effect of mast cells may be mediated through their stimulatory roles in the proliferation of cholangiocytes and in the further activation of HSCs (Meurer et al., 2020). Specific inhibition of mast cells may therefore be another target of intervention to prevent fibrosis. Yet, the reported beneficial effects of mast cells brought discrepancies in this context (Bradding and Pejler, 2018). A complex combination of cells of activation phases may be formed by hepatic macrophages that are classified as residential macrophages (Kupffer cells) and monocyte-derived macrophages (MoMF). Cell sorting implementations could separate these cells (Qiu et al., 2019). For instance, single cell RNA sequencing successfully identified several subpopulations of HSCs (Krenkel et al., 2019). The aforementioned quiescent HSCs generate a homogenous community categorized by high platelet-derived growth factor receptor beta (PDGFR-β) expression. Activated HSCs or myofibroblasts (MFBs) are consequently subdivided into populations expressing collagens, alpha-smooth muscle actin (α-SMA), and/or immune-related markers. MFBs express S100 calcium binding protein A6 (S100A6) as a general marker (Krenkel et al., 2019). The transdifferentiation of quiescent and vitamin A sorting HSCs in MFBs, leading to cell proliferation and collagen expression, is a fundamental step in the process of fibrogenesis (Trautwein et al., 2015). Extracellular signals derived from different cell types such as macrophages or hepatocytes can result in the activation of HSCs. Subsequently, the activation of HSCs was attributed to different cellular processes such as autophagy, oxidative stress, endoplasmic reticulum stress, and disturbances in metabolic pathways (Fan et al., 2020). The bidirectional cascade of fibrosis was evidenced in several studies regarding the regression of fibrosis (Parola and Pinzani, 2019). Since HSCs are responsible for excessive matrix generation, they could also be considered as valuable targets against fibrosis. Blocking the production of collagen types by specific inhibitors was suggested as novel therapeutical options (Gonçalves da Silva et al., 2021). It has been reported that, if the inflammatory is prevented, the anti-inflammatory and restorative functions of macrophages persevere and influence the hepatic niche (Novo et al., 2020). MFBs could become senescent, turn into quiescent-resembled HSCs or be eliminated via cell death processes during the regression of fibrosis (Okina et al., 2020). Certain types of macrophages produce matrix metalloproteinases (MMPs) in order to degrade the excessive ECM.

Currently, there are few therapeutic opportunities for liver fibrosis and the introduction of efficient options needs to be addressed. Suppression of viruses, dietary alterations, decreasing body weight in NAFLD, and induction of fibrosis regression are proposed to stop the disease progression (Kohla et al., 2020; Sheka et al., 2020; Tandon and Berzigotti, 2020). The current clinical trials mostly did not focus on the direct modification of the mechanisms of the disease progressions but investigated other mechanistic approaches such as the causative agents in the underlying liver damage, the received signals from other organs, metabolic pathways, immune system, and cell death processes. Hepatocyte death is considered a key mechanism of liver fibrosis (An et al., 2020). Therefore, prohibition of cell death was proposed as a potential treatment option (Schuster-Gaul et al., 2020). Cell death is classified as necrosis or apoptosis. Necrosis itself was categorized in necroptosis, autophagy, or ferroptosis (Aizawa et al., 2020). However, a general blockage of cell death may be challenging since apoptosis is involved in the regression of fibrosis (Aizawa et al., 2020; Chang and Li, 2020). Moreover, induction of cell death is needed to enhance the death of tumor cells. Cancerous cell overcome apoptosis during oncogenesis processes, helping them to escape the immune system.

Nonetheless, several drugs were introduced to manage liver fibrosis, with the goal to specifically target hepatocyte cells. Oxidative stress stimulates the activity of the apoptosis-signal-regulating kinase (ASK1) which in turn increases hepatotoxicity, inflammation, and fibrosis (Loomba et al., 2018). Selonsertib (GS-4997, Gilead) an ASK1 inhibitor was investigated in NASH patients but the results were not fully satisfactory. Obeticholic acid (OCA) and elafibranor (ELA) are other proposed drugs which target the metabolism in the liver. OCA, a semi-synthetic bile acid was formerly approved in treating primary biliary cholangitis (PBC) and induces the activation of the nuclear bile acid receptor farnesoid X receptor (FXR) which in turn results in attenuation of bile acids. The drug has a significant effect on FXR (>100-fold) compared with endogenous bile acid, decreasing the generation of bile acid and the uptake of lipids and glucose from food (Fickert et al., 2009). ELA as an insulin sensitizer induces the activation of peroxisome-proliferator-activated receptors (PPAR) and increases the functional of endogenous insulin. It also prohibits steatosis via prevention of fat deposition in liver cells and eliminates glucose from the peripheral blood. Current research mostly aims to use a combination of OCA/ELA in the treatment of liver fibrosis (Roth et al., 2019). Cenicriviroc (CVC) is another drug suggested for the treatment of liver fibrosis but its mechanism of action consists in the inhibition of chemokines including CCR2 and CCR5 and thus in preventing lymphocyte and monocyte recruitment. The anti-fibrotic function of CVC was observed *in vivo* (Lefebvre et al., 2016). The effect of an oral drug in this context was investigated in a clinical study referred to as CENATUR (Friedman et al., 2016). The AURORA study is another phase III clinical trial in the treatment of NASH. There are also promising results from phase I and II clinical trials investigating the effects of emricasan, VX-166 from Vertex, and nivocasa. FXR metabolism is also being targeted using drugs such as tropifexor, cilofexor, AKN-083, and INT-767 in different clinical trials. Moreover, lanifibranor and saroglitazar target the PPAR pathway parallel to ELA, but acting on different ligands. Lanifibranor acts on PPARα/γ/δ whereas saroglitazar targets PPARα/γ. Another drug, BMS-986036, a synthetic polyethylene glycolated (PEGylated) fibroblast growth factor 21 (FGF-21), mimics the activity of liver-derived hormone FGF-21 which controls the activation of fatty acids (Fisher et al., 2014). The beneficial effects of insulin modulators such as liraglutide and semaglutide include a decreased insulin resistance and improvement of NASH (Armstrong et al., 2016). Acetyl-coenzyme A carboxylase inhibitors such as GS-0976 or PF-05221304 block the synthesis of lipids in the liver (Harrison et al., 2018). Finally, imitating gut-related signals could be another treatment strategy. NGM282 NGMBio as an analog of gut-derived hormone FGF-19 regulates the synthesis of bile acid, diminishes fat deposition in liver cells, and improves fibrosis (Harrison et al., 2018).

### Importance of HSCs in Liver Fibrosis

Targeted cell-specific therapy was first introduced in the treatment of cancer but met a number of hurdles (Ledford, 2016), and targeting liver fibrosis might also face similar challenges. However, HSCs are definitely key target cells in this context (Josan et al., 2015). Yet, since they are located in the perisinusoidal space, it is difficult to deliver drugs to HSCs. HSCs are activated in response to inflammatory mediators upon liver damage and subsequently differentiate in MFBs (Li et al., 2015). Collagen and other ECM proteins such as MMPs are secreted by activated HSCs, leading to a remodeling of the liver structure (Nielsen et al., 2019). Therefore, inactivation of HSCs is one of the strategic approaches in the introduction of advanced fibrosis restoration. Different receptors such as PPAR (Palomer et al., 2018), platelet-derived growth factor receptor (PDGFR) (Josan et al., 2015), mannose-6-phosphate/insulin-like growth factor II receptor (M6P/IGF-II) (Ungefroren et al., 2018), and integrins (Peng et al., 2016) are expressed on HSCs and may thus be potential targets for pharmacological agents. Targeting M6P/IGFII with human serum albumin consisting of nanoparticles with doxorubicin was reported by Greupink *et al.* (Greupink et al., 2006). However, different side effects of targeting M6P/IGFII shifted the focus toward PDGRR which is significantly upregulated in fibrosis (Greupink et al., 2006). Systemic administration of interferon gamma (IFN-γ) also has some unwanted effects, yet a fusion protein of IFN-γ and PDGFβR bicyclic peptide was shown to inhibit liver fibrosis *in vivo* (Bansal et al., 2014), although a low bioavailability was an important drawback in this intervention. Van Dijke *et al.* (van Dijk et al., 2018) fabricated biodegradable microspheres with the capability of sustain the release of proteins. Integrin proteins that stimulate the activation of the transforming growth factor beta (TGF-β) and thus promote liver fibrosis are considered as potential targeting moieties (Masuzaki et al., 2021). Inhibition of αv integrins by a small molecule (CWHM-12) efficiently decreased lung and liver fibrosis (Santos and Lagares, 2018). In this regard integrin alpha 11 (IαV) is the key isotype of integrins involved in liver fibrosis as it controls the MFB phenotype and fibrogenic pathway, in particular the hedgehog signaling cascade (Bansal et al., 2017). However, integrins are implicated in many other pivotal functions of the body.

The anti-fibrotic function of the relaxin hormone was reported through binding to its receptor peptide 1 family of relaxins (RXFP1) on HSCs (Nikitin et al., 2018). Relaxin can modulate ECM remodeling and thus interfere with the secretion and degradation of its constituents (Palomer et al., 2018). Effects of relaxin on liver fibrosis were documented (Bennett et al., 2017) by preventing fibrosis through induction of an acute alteration in the liver microcirculation confirmed by morphological changes detected in non-parenchymal sinusoidal cells (Feijóo-Bandín et al., 2017). Relaxin can also reduce the content of collagen in the liver (Gracia-Sancho et al., 2015). Yet, relaxin has a short half-life and induces vasodilation upon frequent treatment in chronic fibrosis situation (Ng et al., 2018).

## Magnetic Nanoparticles

**FIGURE 2:**
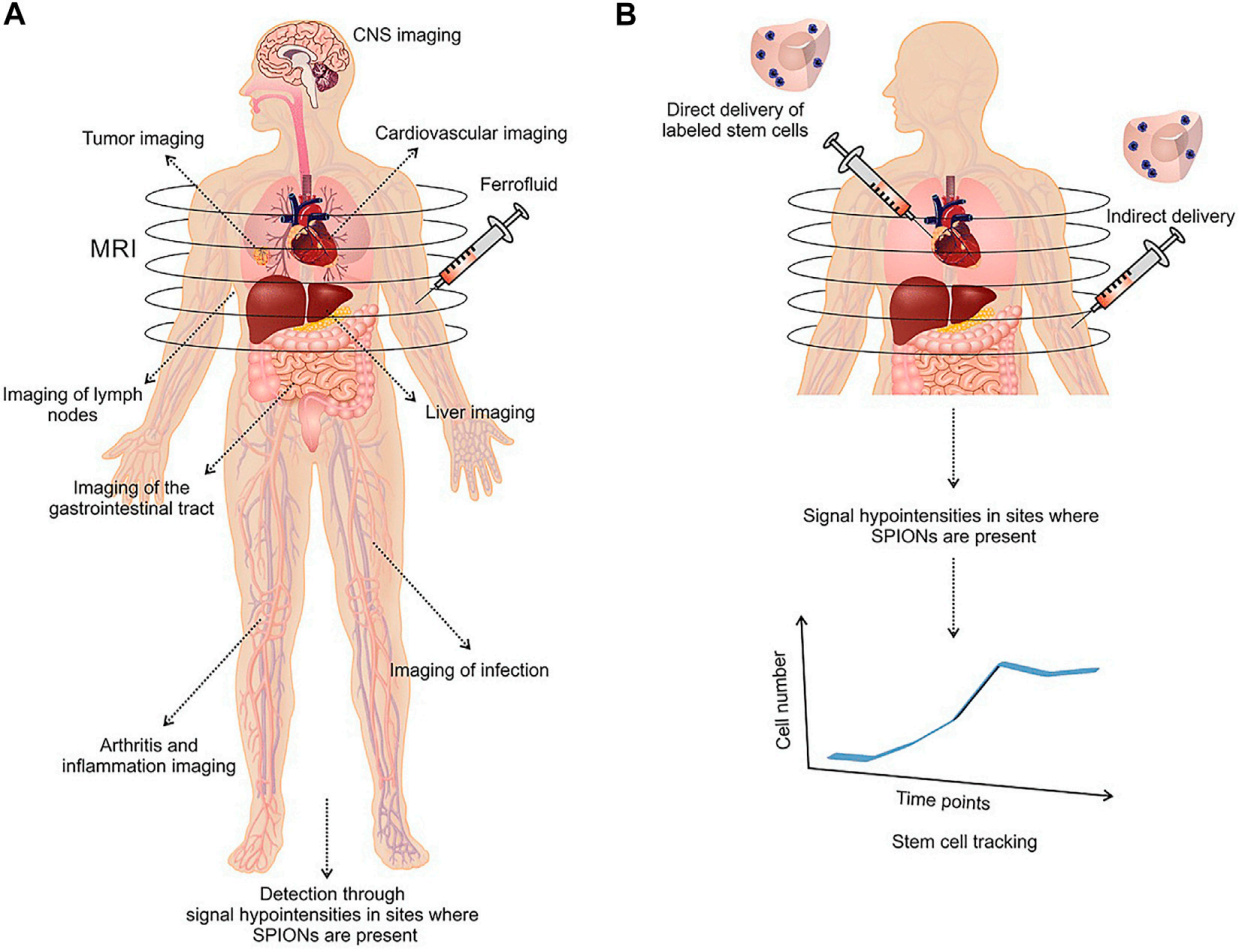
Medical applications of SPION-based MRI in liver, cardiovascular, inflammation, gastrointestinal tract, CNS, and tumor imaging **(A)**. Application of SPION‐labeled stem cells as a monitoring regenerative therapy in tracking of stem cells after transplantation **(B)**. Reproduced with permission (Sharifi et al., 2015) Copyright 2015, Wiley.

Nanomedicine allowed to produce pharmacological agents at nanoscaled dimensions using nanotechnology (Wu et al., 2020). Nanomedicines can be categorized into two major groups based on the nature of the used (organic or inorganic) material. The small size of nanomaterials enables them to easily disperse in aqueous solutions which is important to administer nanodrugs in the peripheral blood (Wu et al., 2020). Nanomaterials possess a high surface-to-volume ratio that provides a large surface with the capability to be functionalized with several ligands which in turn may adapt the pharmacokinetic properties of the material. For instance, the circulation period of nanomaterials may be modified with PEG (Banshoya et al., 2020). Also, specific ligands for cellular receptors provide specific attachment to particular cell types. Conjugation of drugs to the surface of nanoparticles which control on-demand release of the drug is another prominent specification of nanomedicine (Thomas et al., 2010). Additionally, labeled materials may be applied in theranostics for several diseases (Mukherjee et al., 2020). MNPs are specific types of nanomaterials that are highly sensitive to the magnetic field. Interaction of MNPs with the magnetic field which easily penetrates through the human body enables them as valuable biomedical apparatuses. Magnetic Fe_3_O_4_ and γ-Fe_2_O_3_ are the most commonly used MNPs in biomedicine. The cubic inverted spinel construction of bulk magnetite leads to a ferromagnetic behavior of MNPs at room temperature. The nanomagnetite can be rapidly oxidized in maghemite which exhibits similar ferromagnetic specifications and spinel shape with valence electrons. Superparamagnetic iron oxide nanoparticles (SPIONs) are below 30 nm iron-oxide-based MNPs that act in the full name (SPM) regime at room temperature (Angelakeris, 2017). Ultra-small SPIONs (USPIONs) are particles with a size below 10 nm, showing diminished magnetization and elevated anisotropy due to the impact of non-collinear spins at the surface (Angelakeris, 2017). The use of SPIONs and USPIONs in biomedical applications has been in the center of great attention in particular for MRI (Figure 2) (Iv et al., 2015; Sharifi et al., 2015; Hameed and Bhattarai, 2020). The adjustable anisotropy of MNPs is required in distinct applications such as magnetic hyperthermia (Morales et al., 2018). Implementation of variations in the chemical composition of ferrits by doping the spinel shape of ferrite with transitional metals may be performed in order to tune the magnetic anisotropy (Silva et al., 2019).

### Synthesis of Magnetic Nanoparticles

Top-down and bottom-up approaches are important strategies for the generation of MNPs. The top-down method starts using bulk material then processed via different techniques including laser ablation and lithography (Xiao et al., 2017; Jiang et al., 2020). The bottom-up approach starts using metal ions in solution through chemical strategies as the most commonly applied method. Although lithography can successfully control the shape of nanoscaled structures, its application in large-scale production remains challenging (Lan, 2018). The synthesis of complex structures like core-shell MNPs with laser ablation is possible with a broad degree of freedom to control the variations of the material, target, environment, laser regime, and external stimulatory signals (Xiao et al., 2017). Other advantages include the lack of requirement for high pressure, temperature, or organometallic precursors to generate MNPs with high magnetic specification (Svetlichnyi et al., 2017). Therefore, the laser ablation technique is promising for the setting of higher standards in the synthesis of nanomaterials. However, the combination of the aforementioned methods is among the most commonly applied strategies. For instance, the permanent magnet construction is performed with the well-known ball milling technique of MNP synthesis (Yue et al., 2017). It can also be used in the fabrication of MNPs at industrial scale, although it remains arduous to control the size and shape of the particles. Nanoparticle agglomeration is an unwanted effect which might be problematic in biomedical application of nanomaterials (Yue et al., 2017). However, the ball milling apparatus in combination with chemistry could be applied in the mentioned mechanochemical process. Bellusci *et al.* (Bellusci et al., 2018) prepared MNP nanocomposites in a benzene-1,3,5-tricarboxylic acid matrix containing doxorubicin via mechanochemical processing. In this nanocomposite, the high surface area of the porous material enhanced its loading rate and magnetic specifications, permitting the introduction of excellent diagnostic systems. Different steps may be included in the synthesis of typical chemical methods, in particular burst nucleation and a subsequent propagation of nanocrystals, i.e. Ostwald ripening (Sadighian et al., 2020). The size of the particles may be controlled by alterations in temperature, solvents, and other situations as well as the manipulation of the Ostwald process (Sadighian et al., 2020). The resulting nanoparticles must have exact and predefined size, structure, and phase composition (Roca et al., 2019; Salvador et al., 2019). A four-word analysis including strengths, weaknesses, opportunities, and threats (SWOT) was proposed to monitor the common methods to produce nanoparticles (Salvador et al., 2019). The efficiency of co-precipitation, thermal decomposition (high temperature decomposition - HTD), microemulsion, microfluidic synthesis, and dual-particles formation containing different materials was evaluated. Large amounts of MNPs through alkalization of metal salt solution can be obtained using the co-precipitation method. Also, ferrite MNPs or well-crystalized iron oxide were successfully produced with this technique. However, the poor control of size distribution and shape, as well as the reduced quality of the crystals in smaller particles are critical drawbacks of the co-precipitation method (Daffé et al., 2018). High pressure and temperature through hydrothermal routs and solvothermal methods were employed to yield advanced co-precipitation strategies (Huang et al., 2018). Another interesting approach is the cost-effective polyol process that can easily be adjusted to produce large amounts of MNPs with different morphologies (Hemery et al., 2017; Franceschin et al., 2018). On the other hand, the HTD technique may be used to fabricate highly crystalized and narrow size distributed MNPs (Tomitaka et al., 2019) with high degree of magnetization that may be valuable in diagnostic methods (Chen et al., 2018). The limitations of this method according to the SWOT analysis are nevertheless time-consuming processes and expensive protocols (Salvador et al., 2019). Changing the experimental conditions may allow to control the shape of the particles (Salvador et al., 2019). It was shown that cubic MNPs exhibit higher magnetization compared with spherical particles, possibly due to lower levels of disturbed spins on the surface (Noh et al., 2012). Also, cubic MNPs have a higher binding ability resulting from their higher contact area of planar interface (Danai et al., 2021). Additionally, stronger signals are produced via cubic MNPs. Different techniques of co-precipitation as well as HTD are considered to be the most commonly used strategies to produce MNPs (Katz, 2019; Salvador et al., 2019). In addition to being cost-effective and easy, the resultant hydrophilic surfaces allow to functionalize MNPs by co-precipitation. In the second approach, HTD is an efficient technique to produce well-defined shape and narrow size distributed MNPs. However, high cost reagents and lower levels of reaction products with hydrophobic surfaces that provide the possibility of functionalization in further steps both make this technique the currently more attractive approach in the field. Table 1 summarizes the advantages and disadvantages of different synthesis methods of MNPs.

**TABLE 1:** Various methods for magnetic nanoparticles synthesis and their properties.

Synthesis method	Lithography	Laser ablation	Co-precipitation	Thermal decomposition	Hydrothermodynamic	Ball milling
**Advantages**	-Exact control of the shape of nanoparticles	-Good control of external stimulatory signal and other parameters, -No need for high temperature, pressure and precursors	-Simple	-Very good shape control	-Narrow-sized distributed particles	-Applicable for industrial scale
			-Cost-effective	-Very narrow size distribution		
			-High scalable	-High scalable	-Highly crystalized particles	
**Disadvantages**	-Not applicable in large-scale productions	-High cost of laser system	-Poor control of shape	-Complicated synthesis	-Expensive	-Difficult control of size and shape
			-Poor control of size distribution	-High reaction tempretture	-Time-consuming	

### Application of Magnetic Nanoparticles in the Clinic and Perspectives

The most efficient iron-oxide-based drug is the FDA-approved feraheme® (ferumoxytol) in iron-deficient anemic patients and for cases of chronic kidney diseases. Iron is a natural agent that is eliminated from the body via normal metabolic pathways (Toth et al., 2017). The structure of feraheme® consists in non-stoichiometric magnetite MNPs coated with carboxydextran (Iv et al., 2015). Feraheme® is also applied in MRI and magnetic resonance angiography (MRA) as an off-label agent (Hood et al., 2019). The uptake of MNPs via macrophages was studied in high-resolution 3D-maps of pancreatic inflammation *in vivo* (Gaglia et al., 2015). The results indicated that the inflamed lesions of pancreas had higher MNP uptake. The distinct specifications of the inflammatory response on noninvasive imaging should be therefore carefully taken into account. A recent investigation showed the capability of iron oxide to generate reactive oxygen species (ROS) and its implication in the treatment of leukemia (Trujillo-Alonso et al., 2019).

Oral administration of iron oxide in more common compared with intravenous delivery. The low pH level of the gastrointestinal (GI) tract that can degrade iron oxide is still an important barrier against its oral administration (Huang et al., 2015). Coatings may therefore be needed to overcome this problem. Coatings such as silica oxide and gold which are stable in the GI tract could be used for this purpose. Mesenchymal stromal cells (MSCs) provide promising opportunities for the treatment of various disorders including liver fibrosis and HCC (Hu et al., 2019; Kang et al., 2020). Also, MNP-labeled MSCs may be used in diagnostic approaches. Faidah *et al.* (Faidah et al., 2017) showed that the viability (growth) of MSCs was not associated with the use of MNPs in a rat model of cirrhosis. Overexpression of the hepatocyte growth factor (HGF) in MSCs improved the recovery of hepatocytes in a fibrosis model (Lai et al., 2016). Subsequently, labeling of MSCs with MNPs resulted in an increment of cells in MRI-related imaging (Lai et al., 2016), confirming the role of MNPs in the diagnosis of liver fibrosis via MRI. The off-label application of feraheme® is currently growing. The evaluation of the stages of liver fibrosis is a key issue in the diagnosis of patients. Liver biopsy is currently the gold standard method, while imaging strategies such as relaxometry and elastography have not received much attention yet (Guimaraes et al., 2016). It was shown that different MRI-assessable differences are present in patients at different stages of liver fibrosis. However, a dual echo turbo-spin echo method can be used to quantify T2 parameters. Moreover, different stages of liver fibrosis may be distinguished via these techniques (Guimaraes et al., 2016). Iron oxide core nanoparticles can represent the superparamagnetic specifications of T2 or negative contrast agents (Wu et al., 2015). Thus, several types of synthesis, shapes, and nanoparticle coatings with magnetic cores are currently being tested. Nevertheless, there are other obstacles from other perspectives. In a study conducted by Li *et al.* (Li et al., 2018), immunofluorescence markers including indocyanine green were conjugated with Fe_3_O_4_. Then, early staged live fibrosis was detected by targeting the ligand for integrin avb3. Importantly, the accumulation of iron in tissues may become harmful. For instance, it was reported that administration of a single high dose of MNPs induced septic shock response and increased serum levels of liver function enzymes. Also, high doses of MNPs upregulated the expression of genes related to liver cirrhosis compared with normal doses of MNPS (Wei et al., 2016; Zhang et al., 2016). The effects of MNPs on macrophages were also investigated and it was shown that iron overload induces cellular apoptosis via activation of the c-Jun N-terminal kinase (JNK) pathway (Lunov et al., 2010). Iron overload can therefore exerts detrimental effects on the liver that contains large numbers of macrophages.

### Hybrid Magnetic Nanomaterials

**FIGURE 3:**
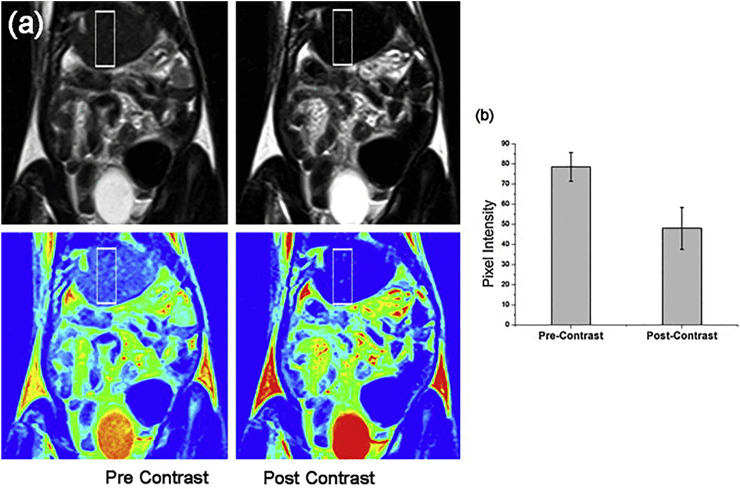
Comparison of pre- and post-contrast *in vivo* MRI of the liver in a region of interest from fibrosed areas (above) and in colorized images of the original MRI (below) after 10 min of application of citrate-coated ultrasmall SPIONs **(A)**. The graph of pixel intensity in pre- and post-contrast of liver is presented in **(B)**. Reproduced with permission (Saraswathy et al., 2014) Copyright 2014, Elsevier B.V.

Although iron oxide displays a moderate toxicity, its biodistribution, biotransformation, and circulation period in blood needs to be monitored (Van de Walle et al., 2020). The hybridization of iron oxide MNPs with different agents such as polymers, noble metals, silica dioxide, and non-magnetic oxides could pave the way to lessen their cytotoxicity (Lavorato et al., 2018; Efremova et al., 2018; Nguyen et al., 2018). PEG was most frequently used as an organic coating for MNPs. Higher molecular weight PEG can significantly increase the blood half-life of MNPs (El-Boubbou, 2018). The pharmacokinetic behavior of MNPs could be substantially affected after coating since their surface charge might be changed (Alphandéry, 2019). A longer half-life is observed in negatively charged MNPs (Alphandéry, 2019). Modification of the surface of MNPs with genes, drugs, and biomolecules may be also via coating. Moreover, hybrid materials exhibit other functionalities, for instance, gold nanoparticles show the surface plasmon resonance phenomenon altering the optical specifications of the material that can be used in photothermal therapy detection (Dheyab et al., 2020). Nanohybrids of gold and magnetite were fabricated in Janus-like MNPs (Efremova et al., 2018). Nanohybrids do not only provide two district surfaces for conjugation of two different molecules but also result in elevated contrast in MRI and real-time delivery of drugs through intravital fluorescence microscopy (Prasad et al., 2018). Citrate-coated ultrasmall SPIONs with highly water dispersibility and smaller size (12 nm) showed acceptable MRI contrast in liver fibrosis imaging and may be applied as platforms for multifunctional nanoprobes in the future. This particle showed good efficacy and biocompatibility in cell labeling and also represented an enhanced MRI contrast in a rat model of liver fibrosis. As shown in Figure 3, the quality of contrast significantly increased upon injection of aforementioned SPIONs (Saraswathy et al., 2014) (Figure 3). Lipid-based nanoparticles have been classified as more prevalent nanomedicines in the market (Ickenstein and Garidel, 2019). Thus, a combination of SPIONs and liposomes offered great advantages in this regard. The use of magnetoliposomes in the induction of nucleic acid release using a magnetic field may also be useful in drug delivery (Dwivedi et al., 2020). The release of DNA from hybrid nanomaterials could be increased via SPIONs (Magro et al., 2017). The increasing number of synthesized hybrid nanomaterials enhances the hope in application of novel means of drug delivery in the future.

## Magnetic Nanoparticles for Liver Fibrosis Theranostics

**FIGURE 4:**
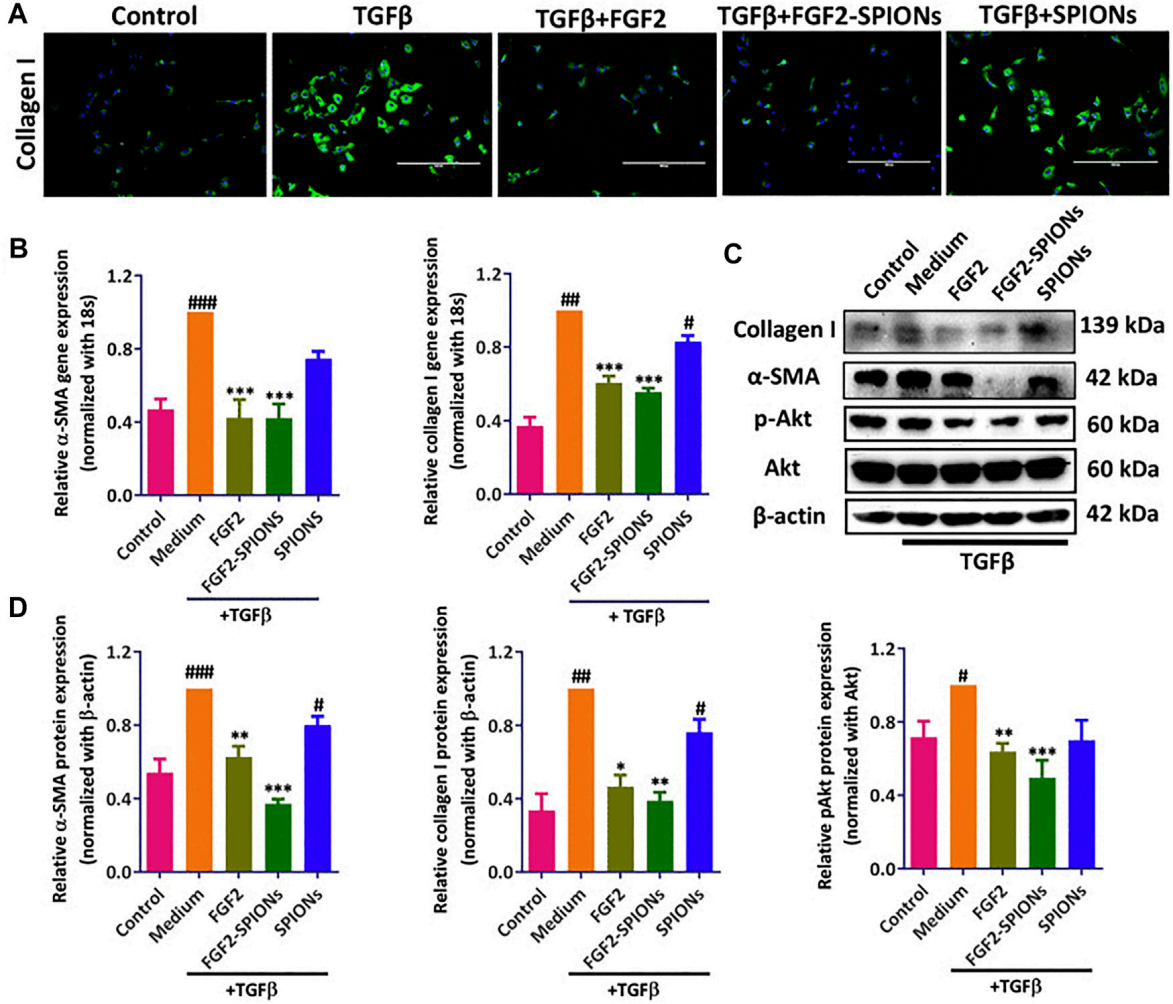
Effects of FGF-2-conjugated SPIONs on HSC activation *in vitro* and *in vivo*. **(A)** Immunofluorescent detection of type-I collagen in control and TGF-β-stimulated LX2 cells in control, FGF-2-, FGF-2-SPION-, or SPION-treated groups. **(B)** Expression levels of *a*-SMA and type-I collagen in control, FGF-2-, FGF-2-SPION-, or SPION-treated groups. **(C)** Western-blot analysis of type-I collagen, pAkt, Akt, *a*-SMA, and *ß*-actin in control, FGF-2-, FGF-2-SPION-, or SPION-treated groups. **(D)** Quantitative analysis of Western-blot results. Reproduced under the terms and conditions of the Creative Commons Attribution 4.0 International License (Kurniawan et al., 2020). Copyright 2020, Elsevier.

The behavior and distribution of nanomaterials are significantly in association with their size. Small particles in the circulation incline towards aggregation or to be decorated with a protein corona and, due to their charge, to produce an electrical double layer (Ramachandran et al., 2020). In this context, dynamic light scattering (DLS) can easily measure the total size of the particles (Ramachandran et al., 2020). Intravenous administration of MNPs leads to the circulation of the particles in the lumen of blood vessels and thus to their interaction with macrophages of the reticuloendothelial system (Alphandéry, 2019). USPIONs exhibit longer in-blood circulation time compared with SPIONs (El-Boubbou, 2018). Also, previous *in vitro*/*in vivo* studies revealed that FGF-2-conjugated SPIONs effectively amended HSC activation as a promising therapeutic method for the treatment of liver fibrosis. This was proved with different molecular experiments. The immunofluorescent images, gene expression and western blot analysis showed FGF2-SPIONs plummeted both TGFβ-induced *a*-SMA and collagen I expression. also, pAkt was downregulated in TGF-b activated LX2 cells (Figure 4) (Kurniawan et al., 2020).

As mentioned above, the size of MNPs may affect their pharmacokinetic properties and biodistribution. MNPs lower than 15 nm are easily filtered by the renal system while MNPs larger than 100 nm accumulate in hepatocytes and those of 100–150 nm are trapped by Kupffer cells and splenic macrophages (Guleria et al., 2018). Spleen and liver as the initial sites of the accumulation of MNPs could be targeted with such particles (Quini et al., 2017). The median size of 10–50 nm is supposed to exert optimal properties, including longer circulation time (Setua et al., 2018). However, other properties such as hydrophobic/hydrophilic features and zeta potential of the particles interfere with their biodistribution (Socoliuc et al., 2020). The MRI enhancement of liver disorders was achieved by a non-specific biodistribution of MNPs (Dadfar et al., 2019). The majority (80%) of non-specific SPIONs which were delivered via an intravenous route were internalized by Kupffer cells (Dadfar et al., 2019). Thus, the produced MR-contrast via MNPs allows to trace hepatic tumor cells (Dadfar et al., 2019).

There are four important non-exhaustive areas where MNPs paly crucial roles. Application of the magnetic field for binding-related cell capturing is the first important concept. Cancer treatment was significantly improved using chimeric antigen receptor (CAR) T cells based on genetic engineering of isolated T cells to form autologous donors that further target specific antigens (Levine, 2015). This technology is currently approved in treating B cell lymphoma, but different clinical studies evaluated its effectiveness against liver cancer (Li et al., 2020). It was shown that macrophages play a chief role in the modulation of adverse effects of CAR T cell-based therapy (Giavridis et al., 2018). The manipulation of other immune cells such as natural killer cells was also addressed (Rezvani et al., 2017). A key technology by cell sorting in closed systems is microfluidics (Liu et al., 2016) using MNPs to provide lab-on-chip devices. Magnetic fields provided powerful systems in tissue engineering processes, in particular the rearrangement of different cell layers (Omelyanchik et al., 2018). Also, establishment of 3D cell culture arrays which enable cellular patterning for an assessment of the impact of fibroblasts and their infiltration properties are provided by magnetic fields (Shim and Hur, 2017). MNPs can be localized in particular parts of the body using magnetic fields which permit to selectively transmit distinct payload to immune cells depending on the phagocytic uptake to injured areas of the liver (Rieck et al., 2019). The spatial concentration of MNPs may also be regulated via MRI apparatuses (Pi et al., 2018).

Mechanical cell control is a second area of application of MNPs. The evaluation of the stiffness of biological molecules and intracellular organelles might be possible in a near future. In this context, magnetic tweezers may be used as potential tools to measure the mechanical forces of biomolecules (Wang et al., 2019). Directed cell apoptosis may be triggered using low-frequency magnetic fields (Zamay et al., 2017). Modulation of stem cell differentiation of the behaviors of single cells was previously regulated in the concept of mechanical cell control (Du et al., 2017). Magnetic tweezer-based manipulations as prominent biophysical methods are useful in single-molecule unfolding, determination of forced-regulated processes in viable cell and rheology measurements (Lipfert et al., 2010).

Drug delivery is a third application of MNPs. In addition to the native toxic effects of MNPs and their thermomagnetic effects, targeted drug delivery with the aid of these particles is a potential means to eliminate injured or infected cells (He et al., 2018). Magnetism-based application in the field of drug delivery has several benefits such as an increased release of drugs from a single carrier from porous materials, an azo-functionalized MNP-based remote control of drug release, and the enhancement of drug release from magnetically regulated nanocarriers (Fuller et al., 2019).

**FIGURE 5:**
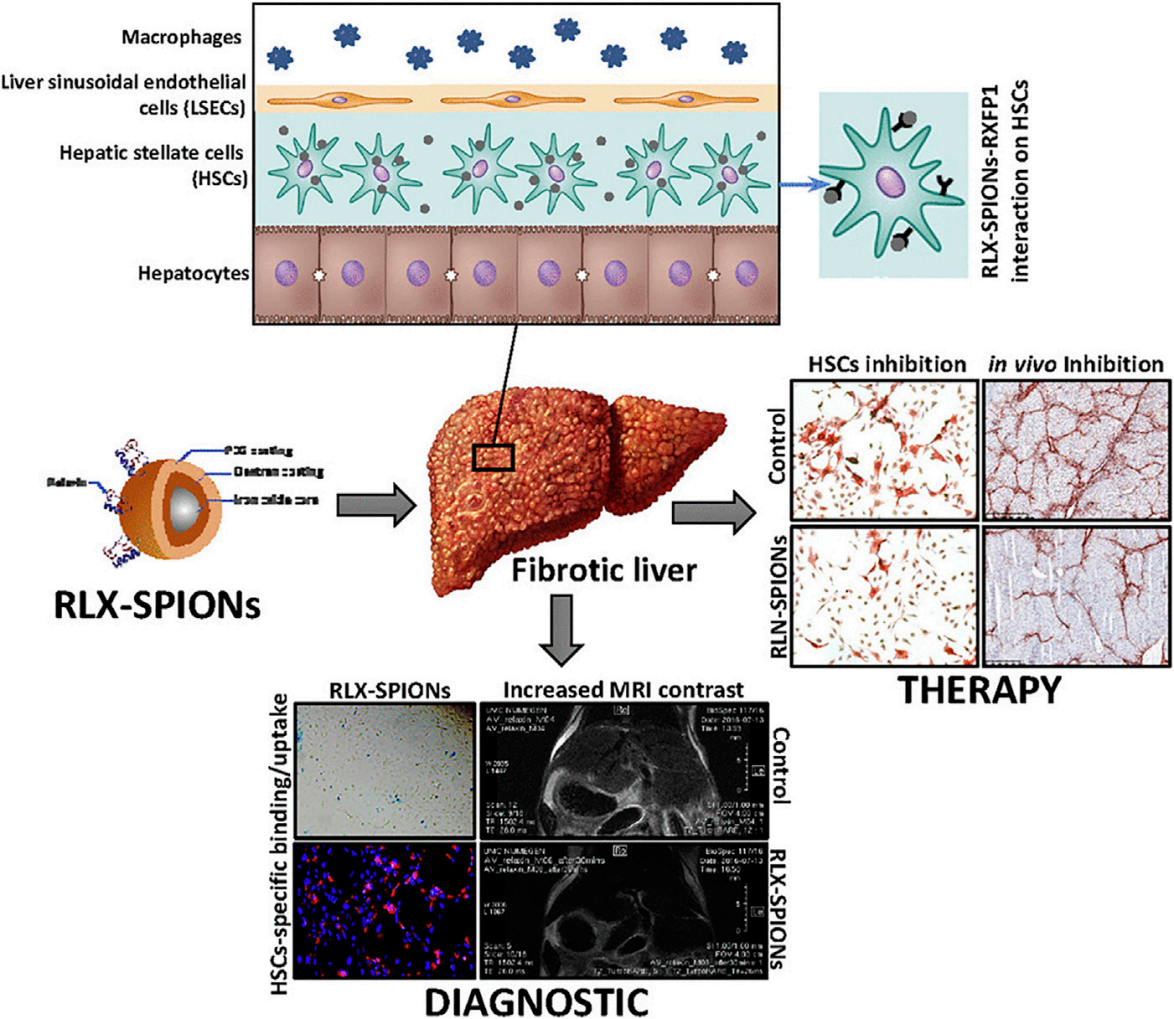
The role of engineered Relaxin in the treatment and diagnosis of liver cirrhosis. Reproduced with permission (Nagórniewicz et al., 2019). Copyright 2019, *Elsevier Inc*.

The fourth application of magnetism is its advantage in imaging techniques. Non-invasive assessment of elastin or collagen in liver fibrosis may be visualized by MRI-based methods (Salarian et al., 2020). Early treatment of liver fibrosis is connected with the early diagnosis of the disease although no established marker of fibrosis on early stages is present. Performing liquid biopsy via detection of N-terminal propeptide of type-III collagen (Pro-C3) might be promising in early diagnosis of liver fibrosis (Piazzolla and Mangia, 2020). Pro-C3 screening was used to detect hepatitis C and to predict the progression of liver fibrosis (Nielsen et al., 2015). Peripheral microRNAs, long non-coding RNA, and microbiomes may be considered as other types of biomarkers (Zhang et al., 2019; Li et al., 2020). The findings of biomarker screening are often confirmed by non-invasive imaging methods. Currently, there is an emerging need for the development of novel techniques in non-invasive diagnosis of liver fibrosis. A targeted USPION-based contrast agent was applied for the MRI-associated detection of fibrosis stages *in vivo* (Saraswathy et al., 2014). The degree of the interactions of protons whose relaxation period changes in the presence of a magnetic field with MNPs determine the MRI contrast level (Gossuin et al., 2016). Moreover, magnetic particle imaging (MPI) is capable of directly imaging the distribution of MNPs. The technique was first represented in 2005 (Nikitin et al., 2018; Sehl et al., 2020). Since MPI exhibits enhanced temporal and spatial resolution compared with MRI, it may represent a promising system for the detection of liver diseases (Sehl et al., 2020). The use of MNPs as biosensors might be another interesting methodology (Gessner et al., 2021). Elastin is increased in later stages of liver fibrosis and imaging probes of elastin can detect late stages of the diseases (Nagórniewicz et al., 2019). USPIONs can bind to the tripeptide arginine-glycine-aspartic acid (RGD) expressed on HSCs and thus, predict liver fibrosis stages via MRI (Zhang et al., 2016). Nanohybrid-based multimodal imaging is another groundbreaking progress to detect liver diseases (Li et al., 2018). The targeting of RGD and fluorescence imaging was increased using USPION-SiO_2_ conjugates with indocyanine green dye. In theranostic applications, a drug should also be used (Bartneck et al., 2012). Relaxin-conjugated MNPs substantially decreased the differentiation, migration, and contractile capability of HSCs (Nagórniewicz et al., 2019). Due to the unique properties of MNPs, their careful application with targeting distinct receptors on HSCs may still potentially pave the way for future adapted treatments against liver fibrosis (Figure 5 ) (Nagórniewicz et al., 2019).

## Outlook and Future Prospects

Unique advantages of Nano medicines as technological drivers of innovation are owed to advanced, targeted, and blood-brain-crossing theranostics platforms. The capability of MNPs to respond external magnetic fields turns them into versatile particles which can be functionalized with bioactive compounds. Diminished magnetization modalities with special surface modifications, the dissociations of functionalized domain with magnetic core, the distances between magnetic field and target site, and potential cytotoxicities are current challenges of magnetic nanotechnology. Importantly, the plausible tissue-toxicities of MNPs should be evaluated. Also, by enhanced nanotoxicology determinations, mechanistic strategies and clinical relevance should be taken into account.

## Conclusion

This review focused on a description of molecular mechanisms and most important factors of deleterious liver diseases such as fibrosis and on MNPs as a promising tool for modern nanomedicine. These nanoscaled materials exhibit a great potential for theranostics of fibrosis due to their unique properties such as good biocompatibility, superparamagneticity, large surface area, high stability, and imaging contrast. Possible complications during the transfer of magnetic nanotechnologies from laboratory models to clinical usage were also discussed.
